# GluN2B mRNA expression and molecular sequence in the brain of pigeons (*Columba livia*)

**DOI:** 10.5455/javar.2025.l909

**Published:** 2025-05-07

**Authors:** Mohammad Rabiul Karim, Ahmed I. Abo-Ahmed, Abu Raihan, Md. Asif Karim Hemel, Md. Alamgir Kobir, Munmun Pervin

**Affiliations:** 1 Department of Anatomy and Histology, Faculty of Veterinary Science, Bangladesh Agricultural University, Mymensingh, Bangladesh; 2 Department of Anatomy and Embryology, Faculty of Veterinary Medicine, Benha University, Moshtohor, Toukh, Egypt; 3 Department of Biochemistry and Molecular Biology, Faculty of Agriculture, Bangladesh Agricultural University, Mymensingh, Bangladesh; 4 Department of Pathology, Faculty of Veterinary Science, Bangladesh Agricultural University, Mymensingh, Bangladesh

**Keywords:** Brain, cDNA, glutamate receptor, GluN2B, PCR, pigeon

## Abstract

**Objectives:**

The current study sought to ascertain the mRNA expression and establish the complementary DNA (cDNA) sequences of pigeon brain’s glutamate receptor 2B of N-methyl-D-aspartate (GluN2B) type.

**Material and Methods:**

Adult pigeons (*Columba livia; n* = 8, sharing an equal number of males and females) were used. After proper anesthesia, the brain was exposed, and small pieces of cerebellum, optic tectum, thalamus, and telencephalon were collected quickly; total ribonucleic acid (RNA) was isolated, and cDNA was synthesized for PCR amplification. The ABI Prism 3100 Genetic Analyzer was used to analyze the sequences of the corresponding cDNA fragments.

**Results:**

In RT-PCR, the findings unequivocally demonstrated that the pigeon brain’s cerebellum, optic tectum, thalamus, and telencephalon all expressed the mRNA for GluN2B. The cDNA sequence of pigeon GluN2B was obtained from PCR-amplified products and included 51 base pairs (bp) of the 5’ untranslated region (UTR), a 4,512-bp open reading frame, and 13 bps of the 3’ UTR. Pigeon GluN2B’s cDNA sequencing displayed 85% identity for human GluN2B and 95% identity for chicken. The amino acid sequences encoded by the pigeon GluN2B gene shared between 85% and 97% similarity with those of humans, rats, and mice. Molecular phylogenetic analysis using the neighbor-joining method showed that pigeon GluN2B is closely related to the GluN2B proteins of these other species.

**Conclusion:**

The findings suggest that certain neurons in the pigeon brain GluN2B mRNA. They also indicate the presence of various glutamatergic networks and connections within the avian brain.

## Introduction

Glutamate and its receptors mediate excitatory neurotransmitters in mammalian brains [1]. Pharmacological studies have confirmed that glutamate functions as a neurotransmitter by using antagonists that inhibit ionotropic glutamate receptors (GluRs). These ionotropic GluRs are widely distributed throughout the brain and are classified according to the specific agonists they bind to, such as AMPA (α-amino-3-hydroxy-5-methyl-4-isoxazole propionic acid), kainate, or N-methyl-D-aspartate (NMDA) receptors [1–3]. In birds, pharmacological studies have provided strong evidence that glutamate is involved in brain functions related to learning, motivation, neural plasticity, memory, and imprinting [4–8]. However, pharmacological evidence has not been able to identify the specific glutamatergic pathways affected using GluR antagonists, as these neural networks have not yet been fully mapped in the avian brain.

*In situ* hybridization studies identified vesicular glutamate transporter 2 (vGluT2) mRNA-expressing glutamatergic neurons (origin of glutamatergic projection) in the olfactory bulb, pallium, thalamus, optic tectum, brain stem, and cerebellar cortex of pigeon and zebra finch [9,10]. They postulated that the distribution of vGluT2 mRNA in birds was like the combined distribution of vesicular glutamate transporter 1 and 2 mRNAs in the brain of mammals [11–14]. Electron microscopical studies showed that immunoreactivity for vesicular glutamate transporter 2 is found in the presynaptic terminals of asymmetric synapses within the septal nuclei, hippocampal formation, and caudal mesopallium in pigeons [15]. The pigeon brain appears to have numerous glutamatergic projections or circuits based on regional variations in glutamatergic neurons and vGluT2 immunohistochemistry.

GluR subunit mRNAs are found in the cell bodies of neurons that receive input from glutamatergic pathways. As a result, the distribution of GluR mRNA may indicate the target regions of glutamatergic neurons within neural networks. In mammals, these mRNAs are broadly distributed across various brain regions [3,16–18]. In avian species, specifically pigeons, studies using mRNA detection and in situ hybridization have mapped the expression of GluA1-4 mRNAs [9,19]. Although a comprehensive map of NMDA receptor mRNA expression in bird brains is still lacking, some research has identified the presence of kainate, AMPA, and NMDA receptors in specific regions like the auditory system and song control centers of adult male zebra finches [10,20], as well as in the auditory ganglia of pigeons [21].

The localization of glutamatergic neurons expressing vGluT2 mRNA in the avian brain was demonstrated in a previous work [9,10], indicating a wide variety of glutamatergic sources.

Glutamatergic neurons are broadly dispersed in the avian brain. The goal of the current work was to identify the complementary DNA (cDNA) sequence of GluN2B and the mRNA expression of this protein in the pigeon brain to evaluate potential targets for glutamatergic neuron projections.

## Material and Methods

### Ethical approval

Animal handling and experimental procedures were carried out following the ethical approval committee’s guidelines (AWEEC/BAU/2023(02)).

### Animals

Mature pigeons (*Columba livia; n* = 8, sharing an equal number of males and females with an average body weight of 302 gm) were used in the current study. Anesthesia of the pigeon was done using sodium pentobarbital (50 mg/kg). The cerebellum, optic tectum, thalamus, and telencephalon were quickly dissected and placed in an RNA stabilization solution (RNAlater, Ambion, Austin, TX, USA) to preserve RNA integrity. The samples were subsequently stored at –60°C until further use.

### Total RNA isolation, cDNA synthesis, and PCR amplification

Total RNA was extracted from pigeon brain regions—including the cerebellum, thalamus, optic tectum, and tel-encephalon-using TRIzol reagent (Invitrogen, Carlsbad, CA, USA). First-strand cDNA was synthesized using the Superscript III First-Strand Synthesis System (Invitrogen). Briefly, a reaction mixture containing 0.5 mM of a 2'-deoxy-ribonucleotide 5'-triphosphate (dNTP) mix, 2.5-μM oligo-dT primer, and 0.5 μg of total RNA was heated to 65°C for 5 min and then immediately cooled on ice. The mixture was then supplemented with the manufacturer-provided reaction buffer, 2 units of RNase inhibitor, 5-mM dithioth-reitol, and 10 units of Superscript III reverse transcriptase, followed by incubation at 50°C for 60 min. To terminate the reaction, the mixture was heated at 70°C for 15 min. The resulting cDNA was stored at –30°C until further use.

In accordance with the manufacturer’s instructions, five hundred nanograms of synthesized cDNA were mixed with Takara Ex *Taq* polymerase (Takara Bio Inc., Tokyo, Japan), the supplied Ex *Taq* buffer, and the included dNTP mix for PCR amplification. Subsequently, 1 μM each of the forward and reverse primers was added to the reaction mixture.

The cDNA sequences of chicken GluN2B (XM_416204), zebra finch GluN2B (XM_002195849), and the partial cDNA sequences of pigeon GluN2B acquired in this investigation served as the basis for the construction of the primers for GluN2B. A positive control, β-actin, was chosen, and considering chicken β-actin (NM_205518) as a model, the primers were developed.

PCR (Table 1) was performed for 35 cycles, with each cycle consisting of a 30-sec denaturation at 94°C, a 40-sec annealing step at 57°C, and a 1-min extension at 72°C, followed by a final extension phase of 5 min at 72°C. The resulting PCR products were purified using the Wizard SV Gel and PCR Clean-Up System (Promega, Madison, WI, USA) before being submitted for sequencing.

**Table 1.** Primers for PCR amplification.

Forward primers	Reverse primers
GluN2B	
5'-tctctcccttgatctgtccg-3' (zebra finch)	5'-ggcttcctggtctgtgtcgt-3' (pigeon)
5'-atgagacagatcccaagagc-3' (chicken)	5'-atggcactgtgtctgtatcc-3' (chicken)
5'-cttacttccctggttaccag-3' (pigeon)	5'-tggtatccgtcctcactgaa-3' (chicken)
5'-acacagcttcatccctgagc-3' (pigeon)	5'-gccatataggcccgtttgt-3' (chicken)
5'-tgattggagaggttgtcacc-3' (pigeon)	5'-tgtgctgccattgggaactg-3' (chicken)
5'-agaggaatatgtggaccagg-3' (pigeon)	5'-ctcaatggccacaccatgaa-3' (chicken)
5'-tgaggttatgagcagccagc-3' (pigeon)	5'-cacagtcatagagcccatcg-3' (chicken)
5'-acctccagctgaaggacagc-3' (pigeon)	5'-gctcattgcttccgccaaac-3' (chicken)
5'-acctgcagaaggatgactcg-3' (pigeon)	5'-ccttccctctcactcaaacg-3' (chicken)
β-actin	
5'-tgcgtgacatcaaggagaag-3' (chicken)	5'-cttctc cttgatgtcacgca-3' (chicken)

### Nucleotide and protein sequence analysis

The sequences of the corresponding cDNA fragments were analyzed using the ABI Prism 3100 Genetic Analyzer (Applied Biosystems, Foster City, CA, USA). The obtained pigeon nucleotide sequences and their predicted amino acid translations were then compared with the GluN2B sequences from other avian and mammalian species (chicken: XM_416204, zebra finch: XM_002195849, rat: NM_012574, mouse: NM_008171, dog: XM_005636716, cattle: NM_001192921, chimpanzee: XM_008969550, and human: NM_000834).

### Image processing

Low-magnification images were obtained using an Epson GT-9300UF scanner (Tokyo, Japan). For high-magnification imaging, a digital camera (Nikon DS-Fi1, Tokyo, Japan) mounted on a light microscope was utilized. The images were then edited for brightness, sharpness, contrast, layout, and text formatting using Adobe Illustrator 10.0J and Adobe Photoshop 7.0J (Tokyo, Japan).

## Results

### Expression of GluN2B in different brain regions based on RT-PCR

**Figure 1.**
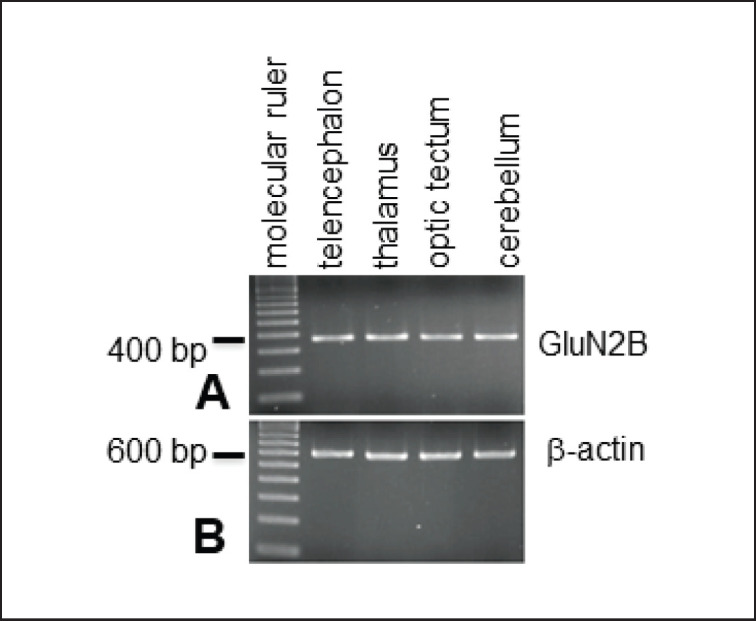
A-B: Detection of pigeon GluN2B mRNA by RT-PCR. Single band (380 bp) in each lane shows the expression of GluN2B mRNA in telencephalon, thalamus, optic tectum, and cerebellum of the pigeon. β-actin (600 bp) is used as a control.

GluN2B expression in the different brain regions was initially examined using the inverse synthesis of RNA and DNA replication (RT-PCR). In RT-PCR, the reaction products from telencephalon, thalamus, optic tectum, and cerebellum showed a single clear band at about 380 bp in each lane by electrophoresis (Fig. 1).

### Pigeon’s brain GluN2B mRNA molecular sequence

A 4,576-base pair (bp) sequence of the pigeon GluN2B gene was obtained through PCR, comprising 51 bp of the 5’ untranslated region (UTR), a 4,512-bp open reading frame (ORF), and 13 bp of the 3’ UTR. This nucleotide sequence was submitted to the GenBank database at the NCBI under the accession number OQ190438.1. The ORF, representing the coding sequence, was translated from the initial methionine (ATG; start codon) to the termination codon (TGA), as documented in the registered sequence for pigeon GluN2B (AC. No OQ190438.1). The obtained pigeon GluN2B (OQ190438.1) ORF cDNA sequences showed 95% identity for chicken (XM_416204.3) and zebra finch (XM_002195849) GluN2B ORF cDNA sequences. The pigeon ORF sequences of GluN2B showed 74% and 75% identity for human (NP_000825.2) and mouse (NP_032197.3) GluN2B ORF cDNA sequences, respectively.

**Figure 2.**
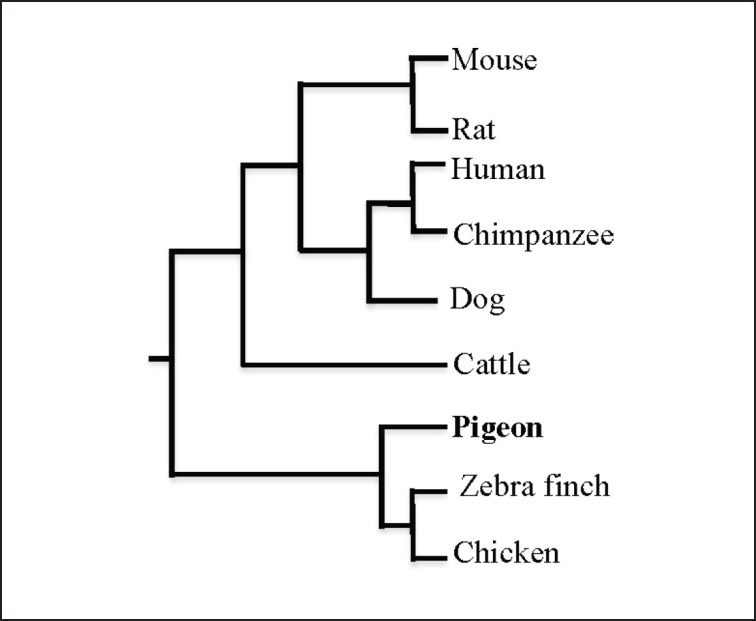
Molecular phylogenetic tree of GluN2B genes in mammals and birds. Phylogenetic trees are inferred from the deduced amino acid sequences using a neighbor-joining (N-J) method by Clustal W alignment [22].

The encoded amino acids of pigeon GluN2B mRNA were 1503, and the amino acid sequence is registered in the GenBank, NCBI, accession number WIG64900.1. The pigeon GluN2B amino acids (WIG64900.1) showed 98% identity for chicken (XP_416204.2) and zebra finch (XP_002195885.1). The sequences of the encoded pigeon amino acids exhibited 91% and 84% similarity with the GluN2B amino acids found in humans (NP_000825.2) and mice (NP_032197.3), respectively. Based on molecular phylogenetic trees (Fig. 2) constructed using the neighbor-joining (N-J) method with Clustal W alignment [22], the GluN2B from the pigeon shows a close evolutionary relationship with GluN2B sequences from other species.

## Discussion

### Expression of GluN2B in different brain regions

Since GluN2B is involved in several human disorders and plays important roles in processes such as memory, learning, pain perception, and eating behavior, it is important to study its location in the brain, its genetic sequence, and the pathways it connects to. NMDA receptors, which include GluN2B, help guide the growth of nerve fibers, support synapse formation, and contribute to brain plasticity during both development and recovery from injury [23]. Studying the structure of the NMDA receptor complex can help us better understand the way the central nervous system works and adapts. The two main types of NMDA receptors in the brain are those that include both GluN2A and GluN2B subunits [24]. It is also believed that NMDA receptors containing the GluN2B subunit—found widely in human and animal neural stem cells—play an important role in spatial memory and the transmission of signals between neurons [25–27].

In our study, GluN2B mRNA was expressed in the pigeon’s cerebellum, optic tectum, thalamus, and telencephalon. On the other hand, its expression is either nonexistent or very low in the cerebellum, optic tectum, hypothalamus, brain stem, and ventral thalamus and moderate in the lateral preoptic region, telencephalon, and dorsal thalamus in adult male zebra finches [28] and in canaries [29]. The vocal learner songbirds exhibit GluN2B mRNA expression in their cerebral vocal nuclei for learned vocalization rather than vocal nonlearners like doves [20]. Specifically, the lateral magnocellular nucleus of the anterior neostriatum—a forebrain region heavily involved in sound production during early vocal learning—shows high levels of GluN2B mRNA expression in birds like zebra finches. However, this expression significantly decreases in adulthood once the learning phase is complete and the song becomes more stereotyped [30,31].

The GluN2B monomer can be observed in neural cells found throughout the animal brain, which develop into granule neurons in the hippocampus [32]. Furthermore, the mouse cerebral cortex expresses the NMDA receptor GluN2B component, which interacts with death-associated protein kinase 1 at extra-synaptic locations; this relationship serves as a crucial mediator for stroke damage [33]. Human brain regions that express GluN2B mRNA include the temporal cortex, hippocampus, putamen, caudate, and nucleus accumbens, in addition to the cerebellum’s molecular layer cells [34].

The pigeon GluN2B mRNA expression in the current study was consistent with its expression in the developing rat brain [35]. However, the hippocampus and cerebral cortex exhibit high levels of postnatal GluN2B expression, while the thalamic nuclei, neostriatum, septum, and cerebellum exhibit moderate levels [35,36]. Furthermore, the rat central amygdala has markedly greater degrees of GluN2B expression than NR2A expression, in contrast to the lateral amygdala [37]. The most recognized NMDA binding component within the framework of the postsynaptic receptor triad is GluN2B, and its expression is primarily extrasynaptic, limited to the spinal cord’s dorsal horn and forebrain regions, and it is thought to be a potential therapeutic target for schizophrenia, stroke, and neurodegenerative illnesses, particularly neuro-pain [38] and Alzheimer’s disease [39].

### Comparison of pigeon NMDA receptor subunit genes with other birds and mammals

In the current study, the obtained full-length coding sequences of the pigeon GluN2B gene shared high homology (80%–95%) with the corresponding GluN2B sequence of chicken and zebra finch, as well as human and mouse. As a result, the pigeon GluN2B mRNA collected for this investigation is unique to the pigeon and homologous to GluN2B in mammals and other birds.

### Possible glutamatergic targets revealed by the GluN2B subunit

The glutamatergic pathways in the brains of birds are not well developed. The avian brain’s glutamatergic functions are not particularly noteworthy. Glutamate appears to be involved in learning, memory formation, persistent potentiation, and excitatory synaptic transmission [5,6]. In neuronal networks, glutamatergic nerves are thought to project onto the expression of mRNAs encoding GluR sub-units. The present investigation found that the telencephalon, the thalamus, the optical tectum, and the cerebellum contain GluN2B mRNAs, indicating the presence of gluta-matergic transmission sites in these areas.

## Conclusion

The entire coding sequence of the pigeon NMDA neurotransmitter channel 2B is a GluN2B gene, and its mRNA expression in the telencephalon-thalamus-optic tectum cerebellum was found in the current study. This pattern of regional GluN2B mRNA expression in the pigeon brain suggests that various areas of the brain include glutamatergic projection sites.

## List of abbreviations

°C, degree Celsius; AMPA, α-amino-3-hydroxy-5-methyl-4-isoxazole propionic acid; cDNA, complementary DNA; GluN2B, 2B of N-methyl-D-aspartate; GluRs; glutamate receptors; gm, gram; kg, kilogram; mg, milligram; min, minute; mRNA, messenger RNA; NMDA, N-methyl-D-aspartate; RNA, ribonucleic acid; sec, seconds; vGluT2, glutamate transporter 2; μg, microgram; μM, micromolar.
